# Association between Periodontitis and Chronic Rhinosinusitis Involving Maxillary Sinus Measured by Lund Mackay Staging System

**DOI:** 10.3390/healthcare10101961

**Published:** 2022-10-07

**Authors:** Khalid Gufran, Abdulaziz Mohammad Alsakr, Abdullah Saad Alqahtani, Nasser Raqe Alqhtani, Dhafer Alasmari, Faisal Fahad Alzamil, Nawaf Munawir Alotaibi, Hamid Mohammed Alhamid, Ashwag Saleem Aldafiri

**Affiliations:** 1 Department of Preventive Dental Sciences, College of Dentistry, Prince Sattam Bin Abdulaziz University, Alkharj 11942, Saudi Arabia; 2 Department of Oral and Maxillofacial Surgery and Diagnostic Science, College of Dentistry, Prince Sattam Bin Abdulaziz University, Alkharj 11942, Saudi Arabia; 3 Department of Periodontology and Oral Medicine, College of Dentistry, Qassim University, Buraydah 52571, Saudi Arabia; 4 College of Dentistry, Prince Sattam Bin Abdulaziz University Alkharj, Alkharj 11942, Saudi Arabia; 5 College of Dentistry, King Saud Bin Abdulaziz University for Health Sciences, Riyadh 14611, Saudi Arabia; 6 College of Dentistry, Riyadh Elm University, Riyadh 13244, Saudi Arabia

**Keywords:** CRS, periodontitis, CBCT, Lund–Mackay system

## Abstract

This study aimed to evaluate the association between periodontitis and chronic rhinosinusitis (CRS) via cone-beam-computed tomography (CBCT) using the Lund–Mackay staging system. CBCT images from different departments of the school of dentistry, at Prince Sattam University were evaluated for the presence of rhinosinusitis. All the CBCT scans were exposed for multiple indications, and no patients had a scan exposed solely for this study. The Lund–Mackay staging system was used to measure the CRS in the CBCT. Descriptive statistics for the frequencies and percentages were used to summarize the data. Logistic regression was used to examine the associations between periodontitis and CRS. Each variable was assessed individually by using multivariable analysis. Collinearity issues among the variables were solved to select a limited set of factors using a stepwise variable selection procedure. A total of 399 CBCT images were included in the current research. Logistic regression showed that only gender was significantly associated (*p* = 0.0001) with the presence of CRS. However, a stepwise variable selection procedure included gender and bone loss as significantly associated with CRS. No significant difference was observed between unilateral vs. bilateral CRS in gender, bone loss, medical status, and periodontitis. However, only gender showed a significant difference in both bilateral vs. no CRS and unilateral vs. no CRS. Periodontitis is not associated with CRS. However, gender has a significant influence on CRS.

## 1. Introduction

Chronic rhinosinusitis (CRS) is a common disease in the upper respiratory tract. The prevalence rate of this chronic condition is 12.5% among the adult US population [1]. The etiology of this phenomenon is multifactorial and ranges from infection caused by microbacteria to iatrogenic origins [2,3,4]. During dental procedures, any foreign body dislocation into the maxillary sinuses might cause iatrogenic sinusitis [5,6,7]. The pathogenesis of the CRS is not very clear, and it is anticipated that mucociliary clearance, abnormalities in the epithelial barrier function, microbiome dysbiosis, and bacterial biofilms might cause this phenomenon [8].

Periodontitis is an inflammatory condition that is concomitant with dental plaque accumulation and characterized by the loss of supporting connective tissue, which gradually damages the tooth-supporting apparatus, specifically the alveolar bone and periodontal ligament [9,10]. Periodontitis is one of the most prevalent conditions and affects 35% of adults [11]. Periodontitis and CRS demonstrate common characteristics, as both are chronic in nature, and stabilization of the polymicrobial biofilms is observed in the oral cavity of periodontitis cases or the airway of CRS cases. Previous studies have endeavored to elucidate the association between periodontitis and CRS by reconciling these characteristics [12,13].

The Lund–Mackay-computed tomography scoring system is commonly used to identify CRS via radiographic and clinical periodontal examination [14]. The magnitude of the pathology is easily determined via computed tomography (CT) images [15]. Changes in the mucosal thickness and air-fluid levels in OMC are the primary findings in CRS. These findings could be observed in several CT systems; however, the Lund–Mackay staging system is frequently used [16]. The opacification level of each sinus, such as maxillary, anterior and posterior ethmoid, sphenoid, and frontal sinuses, is the main determining factor of the Lund–Mackay staging system. A total of 70% of the general population has been reported to have different periodontal diseases [17,18,19,20,21], and radiological signs of sinusitis are directly associated with periodontitis [22]. Moreover, mucosal thickening in the maxillary sinuses observed in radiographs is allied with the periapical lesions [23]. Mucosal cysts and chronic mucosal thickening are more commonly identified in dentate patients over 50 years of age in comparison with the similar edentate control groups [24]. Although chronic periodontitis (CP) and CRS are highly prevalent, a lack of knowledge exists regarding the indisposition of CP and CRS in the general population. Studies found a relation not only between periapical lesions and odontogenic sinusitis but also between periodontal findings via radiographs signifying CRS and CP in general. Thus, this study intended to find whether there an association exists between periodontitis and rhinosinusitis in CBCT imaging measured by the Lund–Mackay staging system.

## 2. Materials and Methods

**Figure 1:**
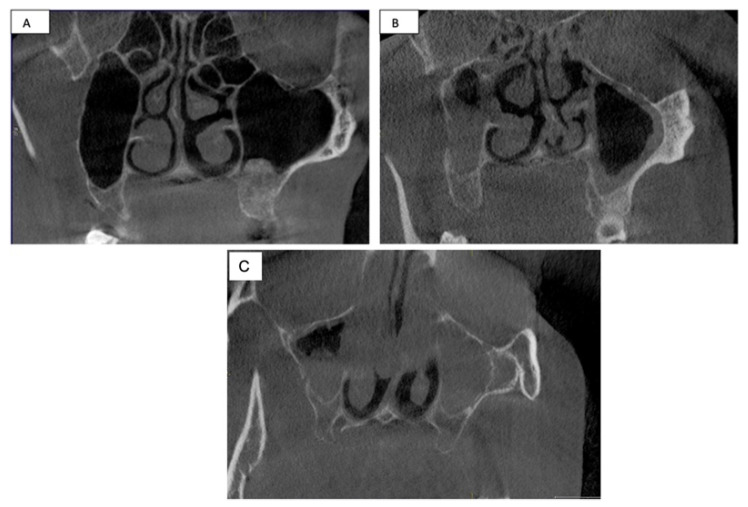
Measurement of CRS via Lund–Mackay staging system. (**A**) Coronal section of CBCT shows a score 0 for both right and left maxillary sinuses. (**B**) Coronal section of CBCT shows a score 1 (partial opacification) for both right and left maxillary sinuses. (**C**) Coronal section of the CBCT shows score 1 in the right maxillary sinus and score 2 (complete opacification) in the left maxillary sinus.

The institutional review board of the Prince Sattam Bin Abdulaziz University approved this study. Cone-beam-computed tomography (CBCT) images from the College of Dentistry Cone Beam Imaging Facility was evaluated for the presence of rhinosinusitis. The included samples consist of scans taken between 1 March 2015 and 29 April 2021. All CBCT scans were prescribed by different departments, including periodontics, oral surgery, oral pathology, and prosthodontics as well as private practices. The CBCT scans were exposed for multiple indications, and no patients had a scan exposed solely for this study. The presence of CRS was measured using the Lund–Mackay staging system. The scoring system was divided into 0 = no opacity, 1 = partial opacity, and 2 = complete opacity (Figure 1).

The following inclusion criteria were followed for this study; patients aged 30 years and above, large- or medium-field-of-view (FOV) CBCT scans that included maxilla or maxilla and mandible, confirmed periodontal diagnosis in the electronic dental record (EDR) that was approved by a faculty member of the department of periodontology. On the other hand, edentulous patients, patients with limited FOV scans, and scans with motion artifacts were excluded from the current study.

### Statistical Analysis

All the statistical analyses were performed in the statistical software IBM SPSS, Version 27 (IBM Co., Armonk, NY, USA). Descriptive statistics for the frequencies and percentages were used to summarize the data. Logistic regression was used to examine the associations between periodontitis and rhinosinusitis. Each variable was assessed individually by using multivariable analysis. Collinearity issues among the variables were solved to select a limited set of factors using a stepwise variable selection procedure. Odds ratios and 95% confidence intervals were calculated by the logistic regression analysis. A *p*-value ≤ 0.05 was considered a significant difference.

## 3. Results

A total of 399 CBCT images were included in the current study. The demographic data from the selected CBCT images include subjects with ages ranging from 30 to 77 years, with a mean age of 47.57 ± 11.59 years. Among 399 CBCT images, 163 were male and 236 were female. A total of 160, 280, and 155 CBCT images displayed bone loss > 50%, CRS, and periodontitis, respectively. CRS was classified using the Lund–Mackay staging system (0 = normal, 1 = partial opacity, 2 = complete opacity), and CBCT images exhibited that only 1.3% and 1.8% were in complete opacity on the right and left sides, respectively. Partial opacity was found in most of the images (Table 1). According to the medical record, 343 of the 399 subjects do not have any medical problems, and the remaining 56 subjects have different systemic diseases, shown in Table 1.

A total of 133 male and 147 female subjects were found to have CRS in the CBCT images. A total of 46.40% of subjects showed >50% bone loss, 44.60% of subjects showed periodontitis, and 15.70% of subjects showed other medical problems. Logistic regression showed that only gender was significantly associated (*p* = 0.0001) with the presence of CRS (Table 2). However, a stepwise variable selection procedure included gender and bone loss as significantly associated with CRS (Table 3).

CRS has been found in three forms—unilateral, bilateral, and no CRS—in the CBCT images; therefore, the generalized logistic model provides a comparison for unilateral vs. bilateral CRS, bilateral vs. no CRS, and unilateral vs. no CRS. No significant difference was observed between unilateral vs. bilateral CRS in gender, bone loss, medical status, or periodontitis. However, only gender showed a significant difference in both bilateral vs. no CRS and unilateral vs. no CRS (Table 4).

## 4. Discussion

CRS is a common phenomenon that has a substantial influence on patients’ quality of life [25]. Due to the proximity of the maxillary teeth to the maxillary sinuses, dental infections cause odontogenic sinusitis (OS), which signifies CRS [26]. As periodontitis is the most common dental infection, the current study aimed to evaluate the association between periodontitis and CRS. A total of 280 CBCT images were identified, with CRS found in 399 images assessed; only 155 images identified periodontitis. Moreover, the current study did not identify any association between periodontitis and CRS.

Very few previous studies have been conducted on CRS and periodontitis. A previous study on the Taiwanese population distinguished an increased risk of periodontitis among patients who had CRS [13]. However, that study only focused on patients who received treatment for periodontitis. This approach is different from that of the current study. Moreover, a Korean study also concluded that patients with CRS are likely to receive treatment for periodontitis, which also contradicts the current research.

Research on gingivitis and mouth breathing demonstrated a significant association in teenage and prepubescent children [27,28]. Moreover, mouth breathing also influenced the CP during scaling and root planning [29]. Dry mouth, which inhibits the biofilm and bacteria washed out by salivation, is related to mouth breathing. Hence, chronic gingivitis eventually leading to CP may appear more in those who are mouth breathers. Therefore, there is a possibility of CRS being associated with CP. However, it is only assumed that alteration of underlying immune function is partially accountable for the association between gingivitis and mouth breathing among adolescents and for enhancing the risk of periodontitis among CRS patients [13,27,28]. Moreover, the different genetic traits may further play important role in the CRS and CP [30,31]. However, the current study did not focus on genetic traits and their effect on CRS and periodontitis did not associate with CRS in the current population.

The current study showed that gender is significantly associated with CRS, which contradicts the outcomes of previous studies [13,32]. Both previous studies were conducted on an East Asian population and the current study was conducted on the southwestern Asian population. The genetic pattern of these two regions is widely different; therefore, the contradiction of the results may be due to the existence of genetic influence. The current study also revealed that 70.18% of CBCT images showed the presence of CRS irrespective of gender. Gender did not show any differences whether the CRS was present unilaterally or bilaterally; significant differences were observed between present or absent CRS regardless of the site.

On the other hand, the presence of bone loss did not associate with CRS during the multivariate analysis. However, the stepwise analysis showed a significant association of CRS with bone loss. A previous study stated that patients who have CRS displayed low bone density and increased risk of osteoporosis [33]. The mucosa has long been thought to be the reason for sinusitis and histopathologic study testified to mucosal changes including congestion, ulceration, edema, submucosal fibroplasia, and submucosal glands [34]. However, a previous animal study showed that mucosal changes due to infection invoked the histopathologic changes in the bone and submucosa, although the microorganisms were confined to the superficial mucosa [35]. Similarly, another animal study on rabbits also stated that CRS could involve bone farther from the primary site of infection. Moreover, infections might extend through the bony structures during the pathogenesis of CRS [36]. These explanations support the finding of our result where bone loss showed significant differences in stepwise analysis.

The current study showed that 86% of the subjects whose CBCT was included were free from other systemic diseases, and only 14% of the subjects had a history of DM, HTN, asthma, hypothyroidism, allergy, cancer, hepatitis, rheumatic fever, kidney problems, thyroid problems, or cholesterol problems. However, the presence of medical illness did not exhibit any association with CRS. Due to the low percentage of systemic diseases and overall non-significant association with CRS, the current study did not evaluate the association of each disease with CRS. A previous study on the Taiwanese population showed a significant association between hypertension, diabetes, hyperlipidemia, and coronary heart disease and CRS [13]. However, the total number of subjects with each disease was larger compared to the current study. In addition, another study showed a significant association between smoking status and the presence of nasal polyps with CRS [32]. However, the current study did not include the smoking status or presence of nasal polyps in this study. The inclusion of smoking status was difficult to include in this study as this was a retrospective study based on the availability of CBCT images. None of the participants in the current study had a history of cardiovascular disease (CVD). The previous study reported that there is a significant association between CP and CVD and recommended that patients with CVD consult dentists and medical practitioners during periodontal treatment [37]. Moreover, poor oral health is also related to amyotrophic lateral sclerosis (ALS); therefore, proper oral health should be maintained for patients with ALS as a multidisciplinary action [38]. In addition, a poor periodontal condition is also strongly associated with different types of cancer, specifically in the organs which are in proximity to the oral cavity, such as the upper gastrointestinal tract and esophagus [39]. Not only are these systematic diseases, but poor oral health along with vitamin D deficiency causes low birth weight and preterm birth [40]. Therefore, poor oral condition could play an imperative role in developing or aggravating life-threatening systematic diseases which should be resolved. These variables might exhibit some insight into association with CRS. Therefore, further prospective and randomized control studies are recommended related to CRS.

## 5. Conclusions

The current study did not find any association between periodontitis and chronic rhinosinusitis. However, gender shows a significant influence on the CRS in the studied population.

## Figures and Tables

**Table 1:** Demographic distribution of the data.

Variables		Frequency	Percentage
Gender			
	Male	163	40.9
	Female	236	59.1
Bone loss > 50%			
	Present	160	40.1
	Absent	239	59.9
Sinusitis			
	Present	280	70.2
	Absent	119	29.8
Sinusitis (right side)			
	Normal	172	43.1
	Partial opacity	222	55.6
	Complete opacity	5	1.3
Sinusitis (left side)			
	Normal	176	44.1
	Partial opacity	216	54.1
	Complete opacity	7	1.8
Periodontitis			
	Present	155	38.8
	Absent	244	61.2
Medical history			
	Cancer	1	0.3
	DM	14	3.5
	HTN	15	3.8
	Asthma	8	2.0
	Hypothyroidism	1	0.3
	kidney problem	1	0.3
	Allergy	1	0.3
	Hepatitis	1	0.3
	DM+ HTN	11	2.8
	DM+ Rheumatic fever	1	0.3
	DM+ HTN+ thyroid+ Cholesterol	1	0.3
	NAD	343	86.0

%, percent; DM, diabetic mellitus; HTN, hypertension; NAD, no abnormality detected.

**Table 2:** Association of CRS and clinical variables.

Variables	CRS Present (*n* = 280)	CRS Absent (*n* = 119)	*p*	OR	CI	
					Lower	Upper
Mean age (SD)	48.89 (11.44)	44.43 (11.39)	0.096	0.980	0.957	1.004
Gender			0.0001 *	0.380	0.233	0.619
Male (%)	133 (47.50)	30 (25.20)				
Female (%)	147 (52.50)	89 (74.80)				
Bone loss > 50%			0.143	0.451	0.156	1.308
Present (%)	130 (46.40)	30 (25.20)				
Absent (%)	150 (53.60)	89 (74.80)				
Medical history			0.531	0.795	0.388	1.628
Present (%)	44 (15.70)	12 (10.10)				
Absent (%)	236 (84.30)	107 (89.90)				
Periodontitis						
Present (%)	125 (44.60)	30 (25.20)	0.886	1.081	0.374	3.125
Absent (%)	155 (55.40)	89 (74.80)				

CRS, chronic rhinosinusitis; *p*, *p*-value; OR, odds ratio; CI, Confidence interval; %, percent; SD, Standard deviation; *, significant difference (≤0.05).

**Table 3:** Association of CRS and clinical variables using a stepwise variables selection procedure.

Variables	CRS Present (*n* = 280)	CRS Absent (*n* = 119)	*p*	OR	CI	
					Lower	Upper
Gender			0.0001 *	0.371	0.229	0.603
Male (%)	133 (47.50)	30 (25.20)				
Female (%)	147 (52.50)	89 (74.80)				
Bone loss > 50%			0.0001 *	0.388	0.239	0.630
Present (%)	130 (46.40)	30 (25.20)				
Absent (%)	150 (53.60)	89 (74.80)				

CRS, chronic rhinosinusitis; *p*, *p*-value; OR, odds ratio; CI, Confidence interval; %, percent; SD, Standard deviation; *, significant difference (≤0.05).

**Table 4:** Analyzing CRS as a three-level outcome (bilateral CRS, unilateral CRS, no CRS) under different characteristics using the generalized logistic model.

Variables	Bilateral CRS vs. Unilateral CRS		Bilateral CRS vs. No CRS		Unilateral CRS vs. No CRS	
	*p*	OR (95% CI)	*p*	OR (95% CI)	*p*	OR (95% CI)
Gender	0.096	0.658 (0.402, 1.077)	0.0001 *	0.314 (0.186, 0.530)	0.011 *	0.476 (0.269, 0.843)
Bone loss > 50%	0.619	1.308 (0.455, 3.762)	0.152	0.430 (0.136, 1.366)	0.079	0.329 (0.095, 1.139)
Medical status	0.988	0.995 (0.505, 1.962)	0.379	0.712 (0.334, 1.516)	0.421	0.716 (0.317, 1.616)
Periodontitis	0.146	0.453 (0.156, 1.316)	0.599	0.733 (0.231, 2.331)	0.453	1.617 (0.461, 5.677)

vs., versus; CRS, chronic rhinosinusitis; *p*, *p*-value; OR, odds ratio; %, percent; CI, confidence interval; *, significant difference (≤0.05).

## Data Availability

Not applicable.
